# Mean platelet volume and coronary plaque vulnerability: an optical coherence tomography study in patients with non-ST-elevation acute coronary syndrome

**DOI:** 10.1186/s12872-019-1115-2

**Published:** 2019-05-29

**Authors:** Jun Wang, Xing Li, Jun Pu, Siyu Jin, Lu Jia, Xiaomei Li, Fen Liu, Yining Yang

**Affiliations:** 1 Department of Coronary Heart Disease, the First Affiliated Hospital of Xinjiang Medical University, Urumqi, 830011 China; 2 The Renji Hospital of Shanghai Jiao Tong University, Shanghai, 200240 China

**Keywords:** Acute coronary syndrome, Thin-cap fibroatheroma, Optical coherence tomography, Mean platelet volume, Plaque rupture

## Abstract

**Background:**

The association between mean platelet volume (MPV) and coronary plaque vulnerability in patients with non-ST-elevation ACS (NSTE-ACS) has not been investigated. We performed a retrospective study to evaluate the association between MPV and plaque vulnerability using optical coherence tomography (OCT).

**Methods:**

Consecutive NSTE-ACS patients who underwent pre-intervention OCT examination in our center were included in this study. Features of coronary plaques in the culprit arteries were classified as rupture, nonrupture with thin-cap fibroatheroma (TCFA), and nonrupture and non-TCFA. ROC analyses were used to determine the predictive efficacy of MPV for plaque rupture, and multivariate logistic regression analysis was performed to evaluate the potential independent predictors of plaque vulnerability.

**Results:**

Overall, 94 patients were included in this study. We identified 17 patients with plaque rupture, 10 with nonrupture with TCFA, and 67 with nonrupture and non-TCFA. ROC analyses showed that MPV ≥ 10.5 fL was predictive of plaque rupture in NSTE-ACS patients. Univariate analyses indicated that patients with higher MPV (≥ 10.5 fL) had higher body mass index and poorer lipid profiles compared to those with lower MPV. Moreover, those with higher MPV had higher incidences of plaque rupture and thrombosis (both *P* < 0.05). Results of multivariate analyses showed that diabetes and higher platelet distribution width (PDW) were independent risk factors of TCFA (*P* = 0.032 and 0.046, respectively), while diabetes, higher BMI, higher PDW, and higher MPV were independent determinants of plaque rupture in our cohorts (P all < 0.05).

**Conclusions:**

Higher MPV is independently associated with higher risk of plaque rupture as evidenced by OCT in our cohort of NSTE-ACS patients.

## Background

Acute coronary syndrome (ACS) is a type of acute coronary artery disease (CAD) that is associated with high morbidity and mortality. ACS is characterized by plaque rupture and acute thrombosis formation in the coronary arteries [1]. Conventionally, plaque rupture and secondary formation of thrombi are considered complex pathophysiological events, and classical CAD risk factors, such as diabetes, smoking, and hypertension, may play important roles in ACS development [2–4]. However, some healthy individuals who do not have the above risk factors can develop ACS [3, 4], suggesting that non-conventional CAD risk factors may underlie ACS pathology. Therefore, identification of novel risk factors for the incidence of plaque rupture in ACS patients is of significance for determining risk stratification and CAD prevention.

Platelets are essential constituents of the blood, and platelet morphology and function play vital roles in the pathogenesis of many diseases related to coagulation, thrombosis, inflammation, and endothelial dysfunction [5]. Under pathological conditions, activated platelets release smooth muscle cell (SMC) proliferation factors, stimulate SMC migration, enhance expression of low-density lipoprotein (LDL) receptors on the surface of fibroblast cell membranes, and activate inflammatory responses via a variety of pro-thrombotic factors, leading to the progression of atherosclerosis [6]. Previous studies suggested that an increase in mean platelet volume (MPV) is an important indicator of platelet activation, which is also closely related to life span, as well as the ultrastructure and functional status of platelets in circulation [7]. Indeed, previous studies mainly focused on the relationship between MPV and CAD risk [8, 9], and confirmed that MPV is a valuable prognostic factor in CAD patients [10, 11]. However, the association between MPV and the risk of acute coronary events, to the best of our knowledge, is under investigated. Particulalry, the risk prediction for patients with non-ST-elevation ACS (NSTE-ACS), including unstable angina and non-ST-elevation myocardial infarction, can be challenging. Using optical coherence tomography (OCT), the optimal intraluminal image tool to evaluate characteristics of coronary plaques, we investigated the potential association between MPV and OCT-evidenced coronary plaque vulnerability in NSTE-ACS patients. Results of our study may be helpful for identifying novel risk factors of plaque rupture and improvement of risk stratification of patients with NSTE-ACS.

## Methods

### Patients and study design

**Fig. 1:**
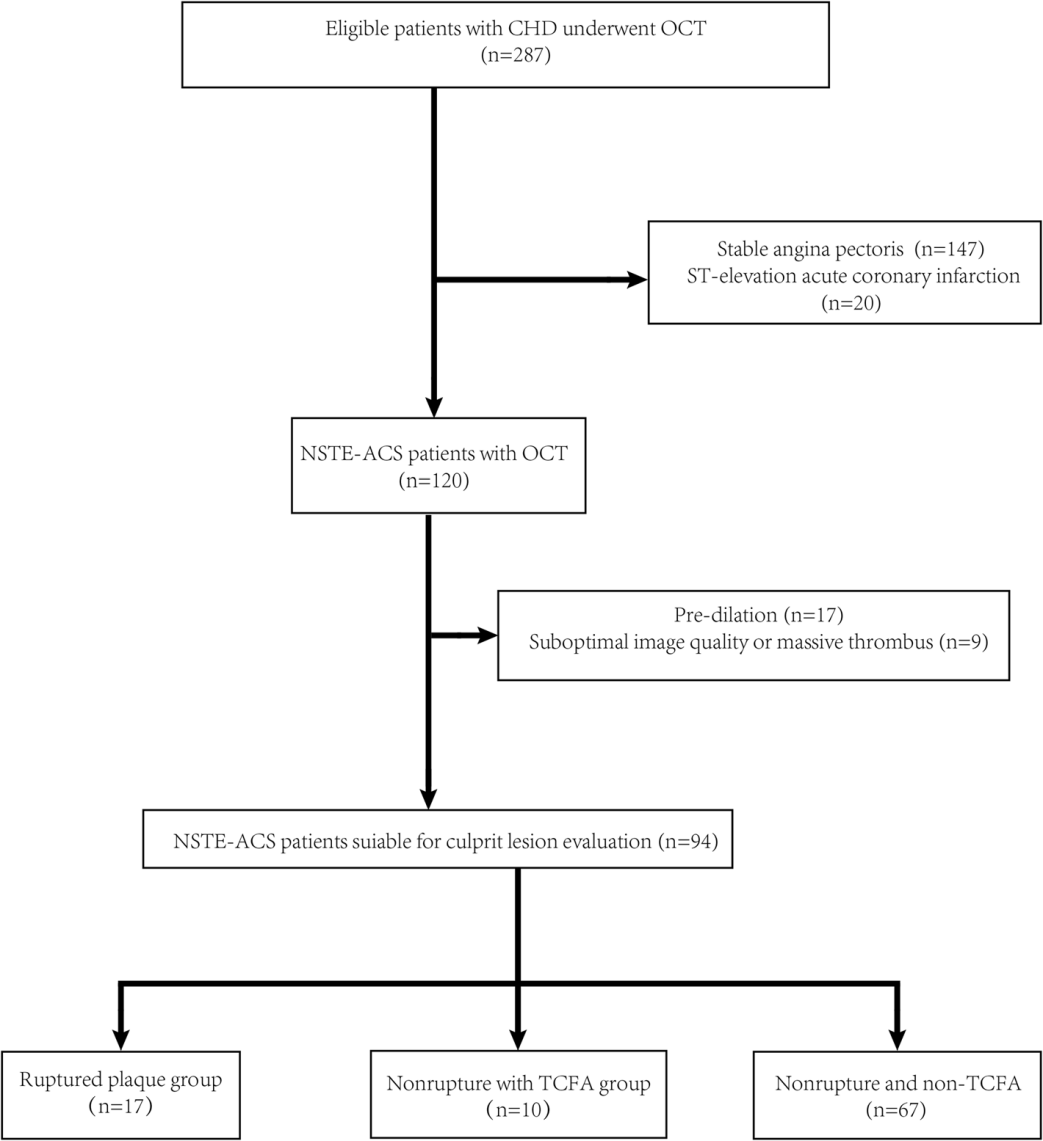
Flowchart of patient inclusion

Patients with NSTE-ACS who underwent pre-intervention OCT examination during coronary angiography (CAG) admitted to the First Affiliated Hospital of Xinjiang Medical University from January 2015 to September 2018 were consecutively screened for study inclusion. NSTE-ACS was diagnosed according to previously established guidelines [12]. The flow chart for patient inclusion and exclusion is shown in Fig. 1. Demographic features, clinical characteristics, CAD risk factors, blood biochemical parameters, echocardiogram (ECG), echocardiography, coronary angiography (CAG), and OCT results were collected. Culprit vessels were determined by CAG, and we focused on plaque vulnerability evidenced by OCT in the culprit vessels. The study protocol was approved by the ethics committee of the First Affiliated Hospital of Xinjiang Medical University before patient enrollment.

### Definitions of CAD risk factors

Hypertension was diagnosed if the patient had a blood pressure ≥ 140/90 mmHg on at least three separate occasions or was being actively treated with antihypertensive drugs [13]. Diabetes mellitus was diagnosed if the patient had a fasting plasma glucose ≥7.1 mmol/L or a two-hour post load glucose ≥11.1 mmol/L [14], and in those with a definite history of diabetes and who were being treated with glucose-lowering agents. The diagnostic criteria for hyperlipidemia were based on the Guideline of Chinese Adult Dyslipidemia Prevention and Treatment (2016) [15]. Body mass index (BMI) was calculated by dividing a patient’s weight in kilograms by the height in meters squared. Smoking was defined as current smoking habit.

### Blood tests

Blood samples from the included patients were drawn immediately after hospital admission,sent immediately for laboratory analysis. MPV was significantly reduced in response to biphasic antiplatelet agents [16, 17]. Therefore, blood collection was performed before the application of an anti-platelet aggregation drug. Blood tests were performed using standard methods in the Central Laboratory of the First Affiliated Hospital of Xinjiang Medical University.

### Coronary angiography and OCT analysis

**Fig. 2:**
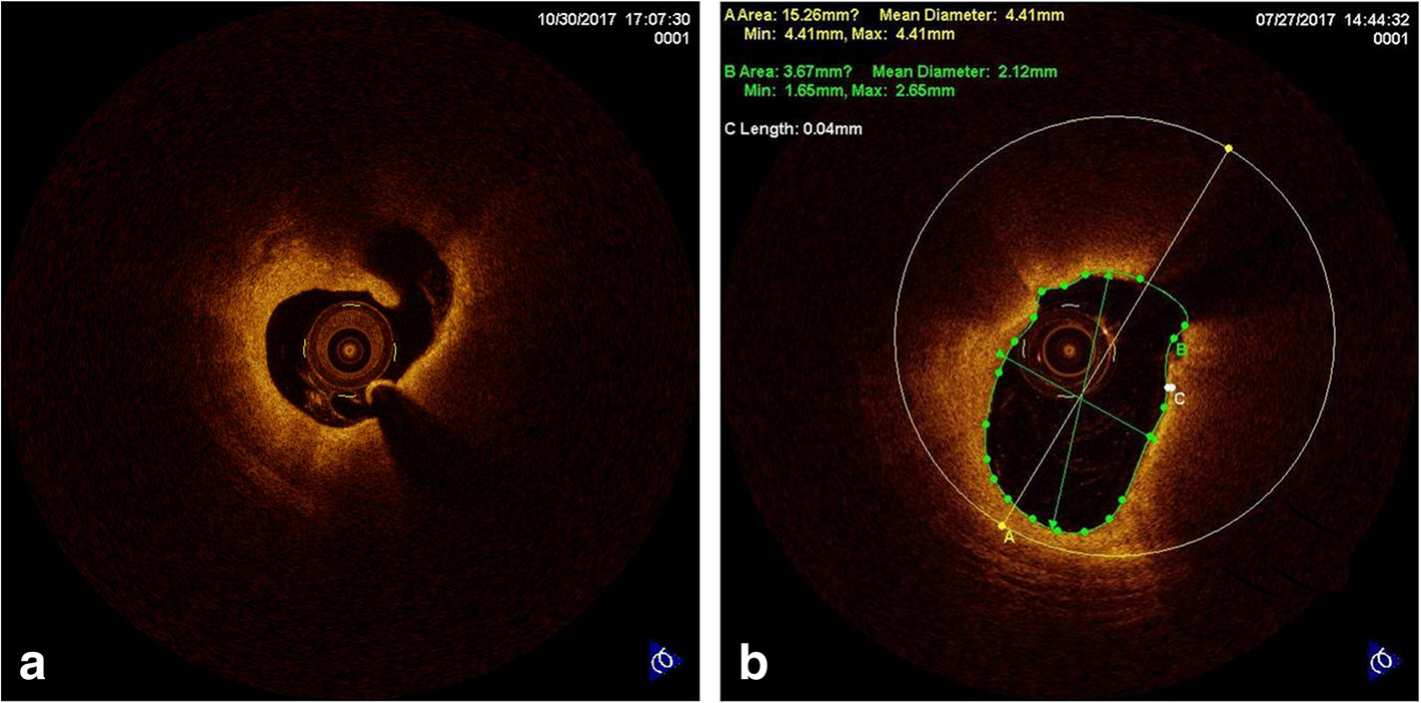
Representative optical coherence tomography images of plaque rupture and thin-cap fibroatheroma. (a) Plaque rupture, (b) Thin-cap fibroatheroma (TCFA)

All patients underwent coronary angiogram within 24 h of admission. All enrolled patients received CAG via a standard method by experienced cardiologists. Quantitative analysis of coronary artery stenosis was determined by experienced interventionists. A commercially available C7-XR OCT intravascular imaging system (C7-XR TM OCT Intravascular Imaging System, St. Jude Medical, St. Paul, MN, USA) was used for OCT examination. OCT images were analyzed based on established OCT diagnostic criteria. Specifically, plaque rupture was identified by fibrous cap discontinuity with a cavity formed inside the plaque (Fig. 2a) [18, 19]. Thin-cap fibroatheroma (TCFA) was defined as a plaque with a maximal lipid arc > 90° and the thinnest fibrous cap thickness was ≤65 μm (Fig. 2b) [18]. Two independent investigators (J.L and S. C.F) who were blinded to clinical angiographic data analyzed the OCT images and the laboratory data using a dedicated off-line review system (St. Jude Medical) at the core laboratory (Xinjiang Medical University). Disagreements were solved by consensus with a third investigator.

### Statistical analysis

All analyses were performed using SPSS 23.0 for Windows statistical software (SPSS Inc., Chicago, IL, USA). Continuous variables are expressed as means and standard deviations, whereas categorical variables are presented as percentages. The Chi-square (χ^2^) test was used for comparing categorical variables. Significant variables in univariate analysis were subsequently included in the multivariate logistic analysis. A *P* value < 0.05 was considered statistically significant.

## Results

### ROC analyses for the association between MPV and plaque rupture

**Fig. 3:**
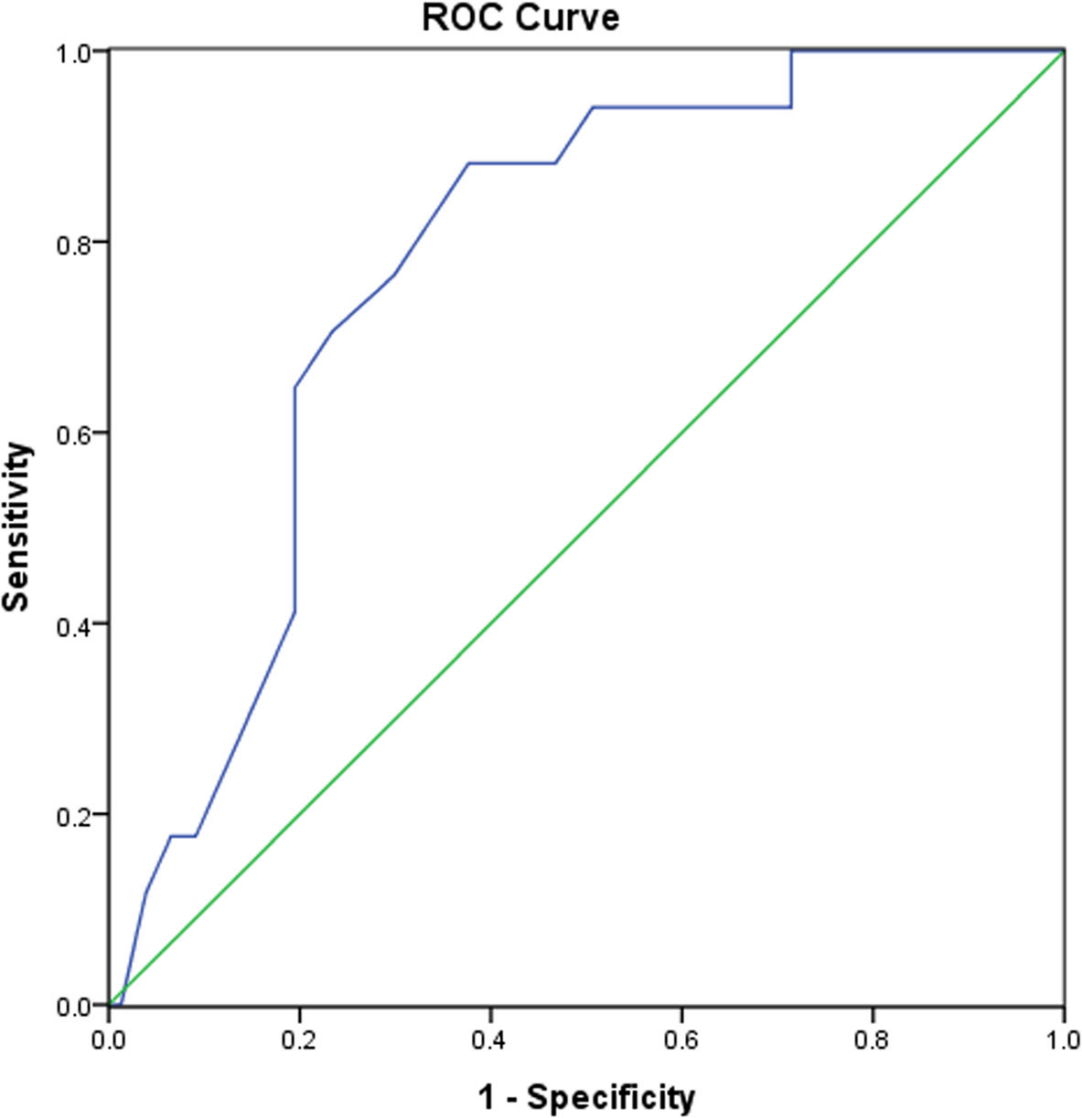
ROC analyses for the predictive efficacy of MPV for plaque rupture

Overall, 94 patients with NSTE-ACS were included in this study, including 33 with non-ST-segment-elevation acute myocardial infarction, and 61 with unstable angina pectoris. OCT analyses for the culprit lesions indicated that 17 patients had plaque rupture, 10 had nonrupture with TCFA, and 67 had nonrupture and non-TCFA. ROC curve analysis showed that MPV was predictive of plaque rupture shown in Fig. 3, and a cut-off value of 10.5 fL of MPV conferred a sensibility of 88.2% and a specificity of 62.3%. The area under the ROC was 0.776, indicating good validity (*P* < 0.001, 95% confidence interval (CI): 0.671–0.880).

### Patient characteristics according to MPV levels

Patient characteristics according to MPV levels are presented in Table 1. Those with higher MPV (≥ 10.5 fL) had higher BMI and poorer lipid profiles compared to those with lower MPV levels. No other variables were significantly different with regards to MPV levels.

**Table 1:** Patient characteristics according to baseline MPV levels

	MPV<10.5 fL	MPV ≥10.5 fL)	t/χ^2^	P
Male	33 (70.2)	38 (80.9)	1.439	0.230
Age (year)	55.04 ± 13.13	56.60 ± 9.42	0.659	0.512
Hypertension	19 (40.4)	26 (55.3)	2.089	0.148
Diabetes mellitus	14 (29.8)	23 (48.9)	3.610	0.057
Smoking	27 (57.4)	30 (63.8)	0.401	0.527
Drinking	6 (12.8)	12 (25.5)	2.474	0.116
Family history of CAD	6 (12.8)	10 (21.3)	1.205	0.272
Previous myocardial infarction	8 (17.0)	7 (14.9)	0.079	0.778
Previous PCI	7 (14.9)	14 (29.8)	3.005	0.083
BMI	24.40 ± 3.2	25.92 ± 3.92	2.052	0.043
HDL-c (mmol/l)	1.03 ± 0.25	0.92 ± 0.25	2.149	0.034
LDL-c (mmol/l)	2.28 ± 0.8	2.63 ± 0.84	2.075	0.041
TC (mmol/l)	3.63 ± 0.92	3.75 ± 1.1	0.560	0.577
TG (mmol/l)	1.81 ± 0.93	1.8 ± 0.94	0.069	0.946
ApoA1 (g/L)	1.14 ± 0.25	1.03 ± 0.28	2.118	0.037
ApoB (g/L)	0.71 ± 0.17	0.86 ± 0.21	3.722	< 0.001
Lp(a) (g/L)	191 (100,292)	205 (98,363)	0.502	0.616
Creatinine (μmol/L)	76.30 ± 18.66	80.72 ± 18.5	1.152	0.252
Carbamide (mmol/l)	5.23 ± 1.58	5.58 ± 1.73	1.016	0.312
eGFR	114.17 ± 36.99	101.1 ± 35.68	1.744	0.085
Uric acid (μmol/L)	315.93 ± 103.6	348.14 ± 83.14	1.663	0.100
EF	60.68 ± 8.05	59.71 ± 8.84	0.546	0.586
HbA1c (mmol/l)	7.23 ± 1.34	7.24 ± 1.4	0.048	0.962
Clinical Diagnosis			0.047	0.829
UAP	31 (66.0)	30 (63.8)		
NSTEMI	16 (34.0)	17 (36.2)		
Medications				
Aspirin	37 (78.7)	32 (68.1)	1.362	0.243
Statins	11 (23.4)	16 (34.0)	1.299	0.254
β-Blockers	16 (34.0)	16 (34.0)	0.000	1.000
ACEI/ARB	14 (29.8)	22 (46.8)	2.881	0.090
CCB	11 (23.4)	11 (23.4)	0.000	1.000
GRACE risk score	102.07 ± 31.11	109.03 ± 21.24	1.001	0.321

*BPC* blood platelet count, *MPV* mean platelet volume, *PCT* thrombocytocrit, *PDW* platelet distribution width, *RBC* red blood cell, *PLT* blood platelet, *HGB* hemoglobin, *HCT* hematocrit, *TBil* total bilirubin, *DBil* direct bilirubin, *Cr* creatinine, *TC* total cholesterol, *TG* triglyceride, *HDL-c* high-density lipoprotein cholesterol, *LDL-c* low-density lipoprotein-cholesterol, *apo-AI* Apolipoprotein A1, *apo-B* Apolipoprotein B, Lp(a), Lipoprotein (a);CCB, calcium channel blockers,

*ACEI* angiotensin-converting enzyme inhibitors, *ARB* angiotensin receptor blocker

### CAG and OCT findings according to MPV levels

CAG and OCT findings of the included NSTE-ACS patients according to the MPV are presented in Table 2. The prevalence of plaque rupture and thrombosis was higher in the patients with higher MPV (≥ 10.5 fL) compared to those with lower MPV levels (*P* < 0.001 and *P* = 0.002, respectively). There was no significant difference in other CAG and OCT findings between those with higher and lower MPV levels.

**Table 2:** Angiographic characteristics and OCT findings according to MPV levels

		MPV<10.5 fL	MPV ≥10.5 fL	t/Z/χ^2^	P
FCT (μm)		0.11 (0.04,0.20)	0.08 (0.04,0.12)	1.642	0.101
Lipid arc, degree		100 (0,178)	149 (0,229)	1.192	0.233
Rupture (%)	No	45 (95.7)	32 (68.1)	12.136	< 0.001
	Yes	2 (4.3)	15 (31.9)		
Erosion (%)	No	40 (85.1)	35 (74.5)	1.649	0.199
	Yes	7 (14.9)	12 (25.5)		
Macrophage accumulation	0	27 (57.4)	15 (31.9)	7.718	0.057
	1	10 (21.3)	18 (38.3)		
	2	10 (21.3)	12 (25.5)		
	3	0 (0.0)	1 (2.1)		
	4	0 (0.0)	1 (2.1)		
Vasa vasorum	No	44 (93.6)	43 (91.5)	0.000	1.000
	Yes	3 (6.4)	4 (8.5)		
Thrombus	No	40 (85.1)	26 (55.3)	9.970	0.002
	Yes	7 (14.9)	21 (44.7)		
Calcified nodule	No	45 (95.7)	44 (93.6)	0.000	1.000
	Yes	2 (4.3)	3 (6.4)		
Characteristic of plaque	Lipid	31 (66.0)	33 (70.2)	2.196	0.333
	Calcified	4 (8.5)	7 (14.9)		
	Fibrotic	12 (25.5)	7 (14.9)		
TCFA		45 (95.7)	39 (83.0)	2.798	0.094
		2 (4.3)	8 (17.0)		
NLA (mm^2^)		10.40 ± 3.38	11.09 ± 3.20	0.932	0.354
Diameter stenosis, %		74.32 ± 15.81	75.72 ± 19.51	0.383	0.702
Lesion length		9.13 ± 3.48	9.97 ± 3.94	1.101	0.274
Target vessel	LAD, n (%)	35 (74.5)	35 (74.5)	0.750	0.687
	LCX, n (%)	5 (10.6)	3 (6.4)		
	RCA, n (%)	7 (14.9)	9 (19.1)		
Location of target plaque	Proximal	34 (72.3)	28 (59.6)	2.310	0.276
	Mid	13 (27.7)	18 (38.3)		
	Distal	0 (0.0)	1 (2.1)		
Number of vascular lesions	1	20 (42.6)	25 (53.2)	1.889	0.389
	2	17 (36.2)	11 (23.4)		
	3	10 (21.3)	11 (23.4)		

FCT, fibrous cap thickness; NLA, normal lumen area

### Coronary risk factors and laboratory data according to plaque vulnerability

Coronary risk factors and laboratory data based on plaque vulnerability, including plaque rupture, nonrupture with TCFA, and nonrupture and non-TCFA, are shown in Table 3. Gender, prevalence of diabetes, previous PCI, current smoking, BMI, MPV and platelet distribution width (PDW) (all *P* < 0.05) were statistically different among the groups. Patients with ruptured plaque, or non-rupture with TCFA are more likely to be male, diabetic, previous PCI, BMI, MPV, MPV ≥ 10.5 fL, PDW as compared with those with non-rupture and non-TCFA (P all < 0.05).

**Table 3:** Characteristics of coronary risk factors and laboratory data according to OCT indicated plaque vulnerability

	Ruptured plaque	Nonrupture with TCFA	Nonrupture and non-TCFA	t/Z/χ^2^	P
Male	16 (94.1)	9 (90.0)	46 (68.7)	7.174	0.028
Age	58.94 ± 10.23	50.10 ± 7.48	55.88 ± 11.93	1.938	0.150
Hypertension	10 (58.8)	3 (30.0)	32 (47.8)	2.142	0.343
Diabetes mellitus	12 (70.6)	7 (70.0)	18 (26.9)	15.23	< 0.001
Current smoking	10 (58.8)	7 (70.0)	40 (59.7)	0.428	0.807
Current drinking	4 (23.5)	2 (20.0)	12 (17.9)	0.272	0.873
Family history of CAD	2 (11.8)	1 (10.0)	13 (19.4)	1.023	0.599
Previous myocardial infarction	1 (5.9)	2 (20.0)	12 (17.9)	1.926	0.382
Previous PCI	7 (41.2)	4 (40.0)	10 (14.9)	6.898	0.032
BMI	28.38 ± 3.98	25.94 ± 1.92	24.23 ± 3.27	11.03	< 0.001
LDL-c (mmol/l)	2.53 ± 0.91	2.66 ± 0.78	2.41 ± 0.83	0.458	0.634
HDL-c (mmol/l)	0.93 ± 0.32	0.98 ± 0.23	0.98 ± 0.24	0.252	0.777
ApoA1(g/L)	1.03 ± 0.28	1.18 ± 0.34	1.09 ± 0.25	1.052	0.354
ApoB (g/L)	0.84 ± 0.26	0.78 ± 0.18	0.77 ± 0.19	0.708	0.495
TC (mmol/l)	3.61 ± 0.98	4.09 ± 0.79	3.65 ± 1.04	0.827	0.441
TG (mmol/l)	2.08 ± 1.02	2.09 ± 0.91	1.70 ± 0.90	1.491	0.231
Lp(a) (g/L)	219 (147,358)	134 (75,302)	191 (96,321)	2.141	0.343
Uric Acid (μmol/L)	348.79 ± 76.98	332.42 ± 59.12	327.72 ± 103.26	0.330	0.720
eGFR	112.59 ± 47.06	105.06 ± 22.26	106.76 ± 35.91	0.195	0.823
WBC	7.33 ± 1.46	7.54 ± 2.61	7.59 ± 2.62	0.076	0.927
PLT	223.12 ± 51.27	256.3 ± 114.34	230.84 ± 61.11	0.822	0.443
MPV	11.18 ± 0.69	11.04 ± 0.54	10.20 ± 1.00	10.017	< 0.001
MPV ≥ 10.5 fL	15 (88.2)	8 (80.0)	24 (35.8)	18.929	< 0.001
PCT (%)	0.25 ± 0.06	0.28 ± 0.11	0.23 ± 0.06	2.117	0.126
PDW	15.33 ± 1.2	15.14 ± 1.71	13.02 ± 2.35	10.536	< 0.001
HCT (%)	0.44 ± 0.05	0.44 ± 0.03	0.43 ± 0.04	0.253	0.777
RBC(10^^12^/L)	4.77 ± 0.46	4.86 ± 0.4	4.77 ± 0.48	0.166	0.848
HGB (g/L)	144.35 ± 16.82	146.5 ± 8.77	143.55 ± 16.21	0.157	0.855
EF	59.51 ± 10.15	58.13 ± 12.70	60.69 ± 7.23	0.432	0.651
HbA1c	7.47 ± 1.15	7.05 ± 1.10	7.20 ± 1.45	0.363	0.696
Clinical Diagnosis				0.367	0.832
UAP	12 (70.6)	6 (60.0)	43 (64.2)		
NSTEMI	5 (29.4)	4 (40.0)	24 (35.8)		
Statins	6 (35.3)	4 (40.0)	17 (25.4)	1.303	0.521
Aspirin	11 (64.7)	7 (70.0)	51 (76.1)	0.937	0.626
β-Blockers	7 (41.2)	2 (20.0)	23 (34.3)	1.337	0.512
ARB/ACEI	6 (35.3)	2 (20.0)	28 (41.8)	1.965	0.374
CCB	5 (29.4)	2 (20.0)	15 (22.4)	0.431	0.806

Abbreviations are as in Table 1

### Independent predictors of plaque vulnerability

Significant variables in univariate analysis were subsequently included in the multivariate logistic analysis. Multivariate logistic regression analyses showed that diabetes, higher BMI, higher PDW, and higher MPV (≥ 10.5 fL) were independent predictors of plaque rupture (P all < 0.05; Model 1; Table 4), while diabetes and higher PDW were independent predictors of TCFA (both *P* < 0.05; Model 2; Table 4).

**Table 4:** Association between patient characteristics and the prevalence of plaque vulnerability: results of multivariate logistic regression analysis

Independent variables	Model 1			Model 2		
	P	OR	95%CI	P	OR	95%CI
Diabetes mellitus	0.043	5.242	1.056–26.015	0.032	6.492	1.176–35.849
BMI	0.006	1.450	1.113–1.887	0.358	1.135	0.867–1.485
PDW	0.012	1.999	1.166–3.425	0.046	1.672	1.010–2.768
Gender	0.095	9.288	0.681–126.667	0.299	3.557	0.324–39.002
Previous PCI	0.079	4.347	0.842–22.453	0.172	3.232	0.601–17.372
MPV ≥ 10.5 fL	0.019	10.154	1.467–70.295	0.069	5.611	0.873–36.053

Model 1: plaque rupture; Model 2: TCFA

### CAG and OCT findings according to the vulnerability of the coronary plaques

CAG and OCT findings according to coronary plaque vulnerability, including patients with plaque rupture, nonrupture with TCFA, and nonrupture and non-TCFA, are shown in Table 5. The degree of macrophage accumulation, thrombus, and normal lumen area were significantly different among the three groups (*P* = 0.005, 0.001, and 0.003, respectively). No significant difference was observed for other CAG and OCT characteristics. Specifically, the macrophage accumulation in the plaque rupture group was higher than that in the non-plaque rupture with TCFA group (*P* = 0.005). The incidence rate of thrombus in the plaque rupture group was higher than that in the non-plaque rupture with TCFA group (*P*<0.001). The NLA of the nonrupture with non-plaque rupture with TCFA group was higher than that of the nonrupture and non-TCFA group (*P* = 0.003). The macrophage accumulation of the TCFA group was higher than that of the nonrupture and non-TCFA group (*P* = 0.005). The incidence rate of thrombus of non-TCFA group was higher than that of the nonrupture and non-TCFA group (*P*<0.001). The NLA of the nonrupture with non-plaque rupture with TCFA group was higher than that of the nonrupture and non-TCFA group (*P* = 0.003).

**Table 5:** Coronary angiographic characteristics and OCT findings according to OCT features of the plaques

	Group	Ruptured plaque	Nonrupture with TCFA	Nonrupture and non-TCFA	t/Z/χ^2^	P
Erosion (%)	No	15 (88.2)	5 (50.0)	55 (82.1)	5.465	0.065
	Yes	2 (11.8)	5 (50.0)	12 (17.9)		
Macrophage accumulation	0	3 (17.6)	2 (20.0)	37 (55.2)	19.328	0.005
	1	7 (41.2)	3 (30.0)	18 (26.9)		
	2	6 (35.3)	4 (40.0)	12 (17.9)		
	3	0 (0.0)	1 (10.0)	0 (0.0)		
	4	1 (5.9)	0 (0.0)	1 (1.5)		
Vasa vasorum	No	16 (94.1)	8 (80.0)	63 (94.0)	2.596	0.261
	Yes	1 (5.9)	2 (20.0)	4 (6.0)		
Thrombus	No	4 (23.5)	4 (40.0)	58 (86.6)	29.624	< 0.001
	Yes	13 (76.5)	6 (60.0)	9 (13.4)		
NLA (mm^2^)		13.52 ± 3.18	10.46 ± 2.59	10.16 ± 3.14	6.332	0.003
Rate of stenosis		81.12 ± 15.89	72.50 ± 16.54	73.85 ± 18.16	1.267	0.287
Lesion length		10.21 ± 4.65	9.40 ± 3.37	9.40 ± 3.55	0.325	0.723
Calcified nodule	No	17 (100.0)	10 (100.0)	62 (92.5)	0.979	0.763
	Yes	0 (0.0)	0 (0.0)	5 (7.5)		
Target vessel	LAD	10 (58.8)	8 (80.0)	52 (77.6)	3.396	0.458
	LCX	2 (11.8)	1 (10.0)	5 (7.5)		
	RCA	5 (29.4)	1 (10.0)	10 (14.9)		
Location of target plaque	Pro	10 (58.8)	7 (70.0)	45 (67.2)	4.296	0.439
	Mid	6 (35.3)	3 (30.0)	22 (32.8)		
	Distal	1 (5.9)	0 (0.0)	0 (0.0)		
Number of vascular lesions	1	9 (52.9)	5 (50.0)	31 (46.3)	0.411	0.982
	2	5 (29.4)	3 (30.0)	20 (29.9)		
	3	3 (17.6)	2 (20.0)	16 (23.9)		

Abbreviations are as in Table 1

## Discussion

In this retrospective study we found that the platelet related blood indices MPV and PDW were independently associated with the risk of plaque rupture, while PDW also independently predicted the formation of TCFA in patients with NSTE-ACS. In view of the fact that changes of MPV or PDW reflect changes in platelet functional status, these findings suggest that MPV and PDW may be used as inexpensive markers for risk stratification in NSTE-ACS patients.

### MPV and coronary plaque vulnerability

MPV is the most commonly used indicator of platelet size, which may reflect platelet activation [20]. Studies have demonstrated that an increase in MPV may confer similar risks as smoking and obesity for the incidence and prognosis of myocardial infarction and atherosclerosis [21]. Previous studies evaluating the association between MPV and CAD risk focused on clinical outcomes [22, 23]. Our study, using OCT, the “gold standard” of current vulnerable plaque judgment in vivo, provided the pathophysiological basis underlying the association between MPV and coronary events by showing that MPV is associated with plaque rupture. Higher MPV has also been associated with CAD risk factors such as hypertension, diabetes, old age, obesity, and smoking [24, 25]. In our study, higher MPV levels were associated with obesity and dyslipidemia, which is consistent with previous findings. However, after adjusting for confounding factors, we found that MPV independently predicted plaque rupture as evidenced by multivariate logistic regression analyses. These findings indicate that an incremental increase in MPV reflects platelet activation. Indeed, many recent studies have linked platelet activity to the development and progression of atherosclerosis [26], especially in patients with ACS. MPV has been found to be higher in ACS patients compared to those with stable CAD [27]. Our data also confirmed MPV as an independent predictor of 6-month mortality or nonfatal myocardial infarction in ACS patients [28]. Incorporating MPV in GRACE’s risk score has been suggested to improve the validity of risk stratification in ACS. Patients with high MPV and high troponin levels had a 4.8-fold increased risk of coronary artery disease [29]. However, a previous study suggested that lower MPV levels were associated with the incidence of MACE in patients with stable angina [30]. Taken together, the relationship between changes in platelet volume and the progression of coronary heart disease may be inconsistent in different subtypes of CAD, which should be confirmed in future studies with larger sample sizes.

Our results are consistent with previous experimental findings that demonstrated that large platelets may be more likely activated, leading to platelet adhesion and aggregation, resulting in a pro-atherosclerotic effect [30, 31]. Large platelets are rich in secretory granules and have a stronger metabolism and enzyme activity. Furthermore, increases in platelet volume lead to the release of young platelets in the bone marrow, which are more reactive and exhibit an enhanced pro-thrombotic activity [32]. In contrast, increased inflammatory factors during acute coronary events may further change platelet morphology and reactivity [33], leading to higher MPV levels. The result is a vicious cycle that exacerbates the course of the disease. However, more experiments are needed to explore the molecular mechanism for changes in MPV in patients with NSTE-ACS.

### PDW and coronary plaque vulnerability

PDW refers to the degree of difference in platelet volume, expressed as the degree of variation in single platelet volume. Increased PDW indicates that platelet volume is not uniform and individual volumes vary greatly. When platelets are activated, they undergo morphological changes by forming pseudopodia, leading to changes in PDW. As a result, platelets become larger and reactive, thus increasing PDW. Furthermore, PDW has also been regarded as a more concrete parameter for platelet activation than MPV, as it does not increase during simple platelet swelling [34, 35]. Accordingly, some studies have suggested a potential relationship between PDW and CAD [36, 37]. Our study showed that PDW was an independent risk factor for plaque rupture as well as TCFA occurrence in NSTE-ACS patients. These results may reflect rapid platelet consumption and activation in patients with unstable coronary plaques. Although the accurate mechanism is unclear, our findings support that PDW confers more predicted efficacy to plaque vulnerability in NSTE-ACS patients since it was associated with plaque rupture and the formation of TCFA under OCT. Further studies are needed to confirm our findings and expand the role of PDW in risk stratification in NSTE-ACS patients.

### Study limitations

There are several limitations in this study that should be acknowledged. First, this study is a retrospective observational single-center study, and results of our study should be confirmed with prospective cohort studies. Secondly, we focused on coronary plaques in culprit vessels. Predictors for the plaque composition of non-target lesions were not analyzed. Thirdly, large residual thrombi may blur the lumen boundary and the underlying plaques, making it difficult to assess the underlying plaque characteristics. Finally, this study attached undue importance to qualitative differences of coronary plaques and thus ignored the observation of spatial distribution of different plaques.

## Conclusions

Higher MPV and PDW are independently associated with higher risk of plaque vulnerability, as evidenced by OCT analysis in our cohort of NSTE-ACS patients. Potential uses of MPV and PDW for risk stratification in NSTE-ACS patients deserve further investigation.

## Data Availability

The data that support the findings of this study are available from the First Affiliated Hospital of Xinjiang Medical University but restrictions apply to the availability of these data, which were used under license for the current study, and so are not publicly available. Data are however available from the authors upon reasonable request and with permission of the First Affiliated Hospital of Xinjiang Medical University.

## Abbreviations

ACEI: Angiotensin-converting enzyme inhibitors
ACS: Acute coronary syndrome
apo-AI: Apolipoprotein A1
apo-B: Apolipoprotein B
ARB: Angiotensin receptor blocker
BPC: Blood platelet count
CAG: Coronary angiography
CCB: Calcium channel blockers
Cr: Creatinine
DBil: Direct bilirubin
FCT: Fibrous cap thickness
HCT: Hematocrit
HDL-c: High-density lipoprotein cholesterol
HGB: Hemoglobin
LDL-c: Low-density lipoprotein-cholesterol
Lp(a): Lipoprotein (a)
MPV: Mean platelet volume
NLA: Normal lumen area
NSTE-ACS: Non-ST-elevation ACS
OCT: Optical coherence tomography
PCI: Percutaneous coronary intervention
PCT: Thrombocytocrit
PDW: Platelet distribution width
PLT: Blood platelet
RBC: Red blood cell
SMC: Smooth muscle cell
TBil: Total bilirubin
TC: Total cholesterol
TCFA: Thin-cap fibroatheroma
TG: Triglyceride
